# Prevalence and natural history of and risk factors for subaneurysmal aorta among 65-year-old men

**DOI:** 10.1080/03009734.2019.1648611

**Published:** 2019-08-28

**Authors:** Knut Thorbjørnsen, Sverker Svensjö, Khatereh Djavani Gidlund, Nils-Peter Gilgen, Anders Wanhainen

**Affiliations:** a Department of Surgical Sciences, Section of Vascular Surgery, Uppsala University, Uppsala, Sweden;; b Centre for Research and Development, Uppsala University/County Council of Gävleborg, Gävle, Sweden;; c Department of Surgery, Falun County Hospital, Falun, Sweden;; d Department of Surgery, Eskilstuna County Hospital, Eskilstuna, Sweden

**Keywords:** Abdominal aortic aneurysm, prevention and control, screening, smoking, subaneurysmal aorta, ultrasonography

## Abstract

**Background:** The aims of this study were to determine the prevalence of screening-detected subaneurysmal aorta (SAA), i.e. an aortic diameter of 2.5–2.9 cm, its associated risk factors, and natural history among 65-year-old men.

**Methods:** A total of 14,620 men had their abdominal aortas screened with ultrasound and completed a health questionnaire containing information on smoking habits and medical history. They were categorized based on the aortic diameter: normal aorta (<2.5 cm; *n* = 14,129), SAA (2.5–2.9 cm; *n* = 258), and abdominal aortic aneurysm (AAA) (≥3.0 cm; *n* = 233). The SAA-group was rescanned after 5 years. Associated risk factors were analyzed.

**Results:** The SAA-prevalence was 1.9% (95% confidence interval 1.7%–2.1%), with 57.0% (50.7%–63.3%) expanding to ≥3.0 cm within 5 years. Frequency of smoking, coronary artery disease, hypertension, hyperlipidemia, and claudication were significantly higher in those with SAA and AAA compared to those with normal aortic diameter. Current smoking was the strongest risk factor for SAA (odds ratio [OR] 2.8; *P* < 0.001) and even stronger for AAA (OR 3.6; *P* < 0.001). Men with SAA expanding to AAA within 5 years presented pronounced similarities to AAA at baseline.

**Conclusions:** Men with SAA and AAA presented marked similarities in the risk factor profile. Smoking was the strongest risk factor with an incremental association with disease severity, and disease progression. This indicates that SAA and AAA may have the same pathophysiological origin and that SAA should be considered as an early stage of aneurysm formation. Further research on the cost-effectiveness and potential benefits of surveillance as well as smoking cessation and secondary cardiovascular prevention in this subgroup is warranted.

## Introduction

Screening elderly men with ultrasound (US) for abdominal aortic aneurysm (AAA) is recommended by several randomized controlled trials (RCTs) (1–4). These studies have demonstrated an approximately 50% reduction in AAA mortality from US-based screening. The largest and most influential RCT, the Multicentre Aneurysm Screening Study (MASS), also demonstrated a reduction in all-cause mortality among those who underwent screening (5). Based on these findings, national screening programs have been implemented in Sweden, England, and the United States (6–9). The evidence for AAA screening in women is still insufficient; therefore, population-based AAA screening programs for women are not currently implemented in Sweden (10).

There is an ongoing debate regarding the threshold aortic diameter for continued surveillance (11,12). Most screening programs define the minimum aortic diameter for an aneurysm as ≥3.0 cm and exclude those with aortic diameters below this threshold from further surveillance, whereas some also include individuals with aortic diameters in the range of 2.5–2.9 cm, also called subaneurysmal aortas (SAAs), in the surveillance program (12). The results from several contemporary studies suggest that this subgroup should be classified as an ‘aneurysm in formation’ and should be treated as such (7,10–13).

Observational studies indicate that >50% of those with an SAA develop a true AAA within 5 years after the initial scan (14–16). More importantly, a considerable proportion of these individuals reach the threshold for surgical intervention within 10–15 years of initial screening (15,16) at an age where they could still benefit from elective AAA repair.

The association between AAA and risk factors such as smoking, cardiovascular disease, hypertension, and hyperlipidemia, as well as that between AAA and increased all-cause mortality, is well documented in numerous population-based studies (17–20). There is also evidence indicating that individuals with an aortic diameter between 2.5 cm and 2.9 cm have significantly higher rates of all-cause mortality than those with an aortic diameter <2.5 cm (15,19–24). The risk factors for men with SAA have, however, been rather sparsely documented (15).

With the implementation of large-scale screening programs, an increasing number of subjects with SAA will be detected, and there is clearly a need for more knowledge regarding this subgroup of individuals, as well as regarding the associated risk factors and morbidity.

The aims of this study were to determine the SAA prevalence among 65-year-old men in middle Sweden, to document associated risk factors, and to assess the degree of comorbidities compared with men with AAAs and normal aortas. In addition, we determined the natural history of SAAs after 5 years of surveillance.

## Methods

**Figure 1.**
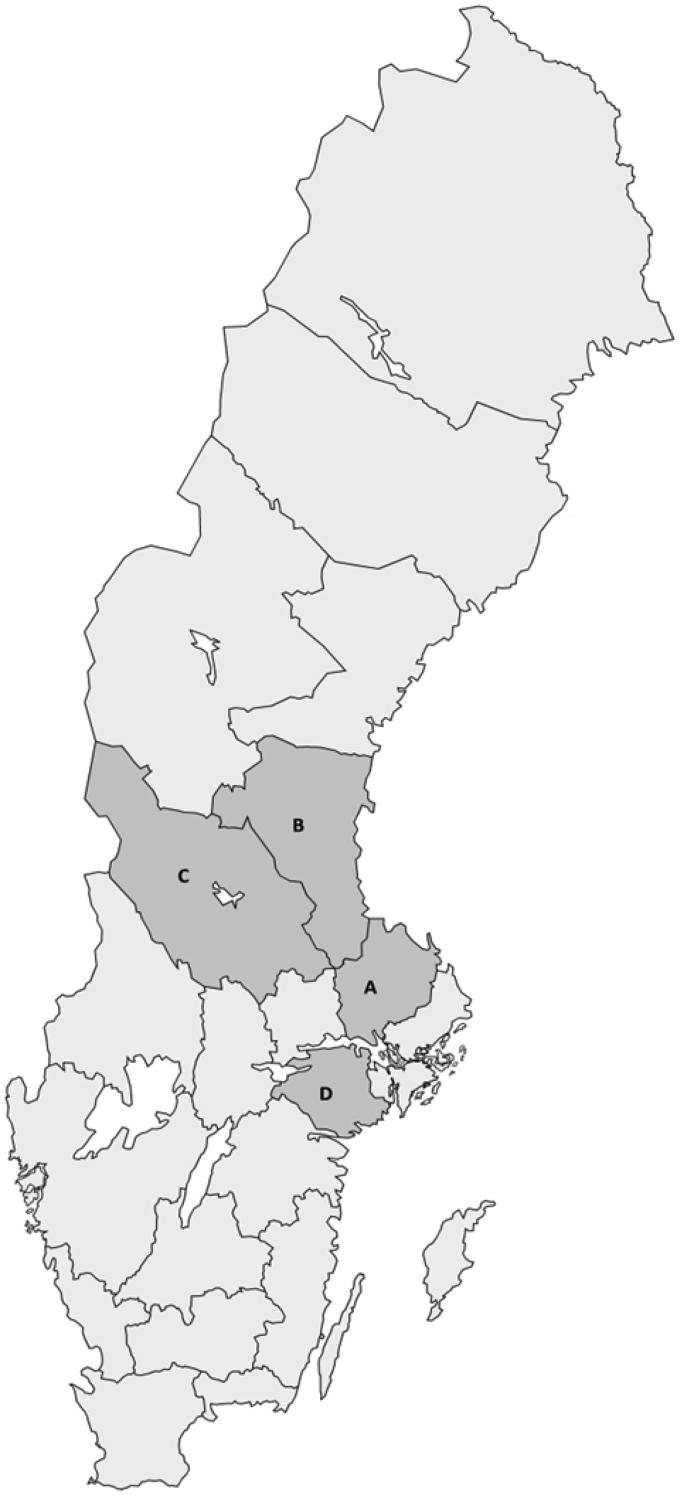
Map of Sweden showing the geographical area of the four counties in middle Sweden. The uptake area comprises: (A) Uppsala, population 367,483; (B) Gävleborg, population 285,452; (C) Dalarna, population 281,046; (D) Sörmland, population 290,711. Population numbers from 2017.

A population-based screening program for AAA among 65-year-old men was introduced in the county of Uppsala in 2006, and similar programs were gradually launched in the neighboring counties of Dalarna, Sörmland, and Gävleborg (Figure 1). Between 2006 and 2010, all 65-year-old men in the four counties were identified through the National Population Registry and invited to undergo an US examination of the abdominal aorta. Subjects with a history of AAA repair or those who were under surveillance for a known AAA were excluded from invitation. No other exclusion criteria were used.

The US examinations were centralized to one hospital in each county with the exception of one county (Gävleborg), where screening was offered at two hospitals. All US examinations were conducted by registered nurses who were specially trained in ultrasonography or ultrasound technicians. All subjects had a single US scan with measurement of the maximum anteroposterior diameter of the infrarenal aorta using the leading-edge-to-leading-edge (LELE) method (25).

The patients were divided into three groups based on the maximum aortic diameter. An AAA was defined as a maximum aortic diameter of ≥3.0 cm, an SAA was defined as a maximum aortic diameter of 2.5–2.9 cm, and a normal aorta was defined as a maximum aortic diameter <2.5 cm.

Of those invited, 14,678 subjects were asked to complete a standardized health questionnaire containing questions regarding first-degree relatives with AAA, family and medical history, smoking status (classified as never, former, and current smokers and smoking duration), coronary heart disease (defined as angina pectoris and/or myocardial infarction), diabetes mellitus (including dietary or medical treatment), cerebrovascular disease (transient ischemic attack or stroke), hypertension, hyperlipidemia, claudication, renal failure, and chronic obstructive pulmonary disease (COPD). Men diagnosed with SAA were followed up at 70 years of age with an US of the abdominal aorta as part of a regional protocol. Eight men underwent a complementary computed tomography (CT) scan because of an incomplete US scan or iliac aneurysms.

Using the Swedish Vascular Registry (Swedvasc), a nationwide registry with high internal and external validity (26), all men who had already been treated for an AAA were identified and excluded. Men under surveillance for a known AAA were identified from local hospital registries.

Statistical analyses were performed with IBM SPSS Statistics software version 24.0 (IBM, Armonk, NY, USA). For comparisons of continuous data, the independent samples *t* test was used. An uncorrected chi-square test was used to compare three proportions according to associated risk factors. Proportions are presented with 95% confidence intervals (CI). To estimate the odds ratio (OR) for risk factors associated with SAA, the variables with *P* < 0.1 in a univariate analysis were entered into a multivariable logistic regression model. A value of *P* < 0.05 was considered statistically significant.

The study was performed according to the principles of the Declaration of Helsinki and was approved by the Ethics Committee of the Uppsala/Örebro Region (Dnr 2006:112 and Dnr 2018/099). As specified by the Ethics Committee, informed consent was not required.

## Results

Between 2006 and 2010, a total of 21,938 men were invited, of whom 18,361 were screened (83.7% attendance). Of those, 18,317 (99.8%) had an appropriate US measurement and were included in the analysis. Twenty-four men were already under surveillance for known AAAs, and 62 living men had previously undergone AAA repair.

A total of 347 SAAs were detected (1.9%; 95% CI, 1.7%–2.1%), and 316 men had an AAA (1.7%; 95% CI, 1.5%–1.9%). A normal aorta was observed in 17,654 men (96.4%; 95% CI, 96.2%–98.6%).

**Figure 2.**
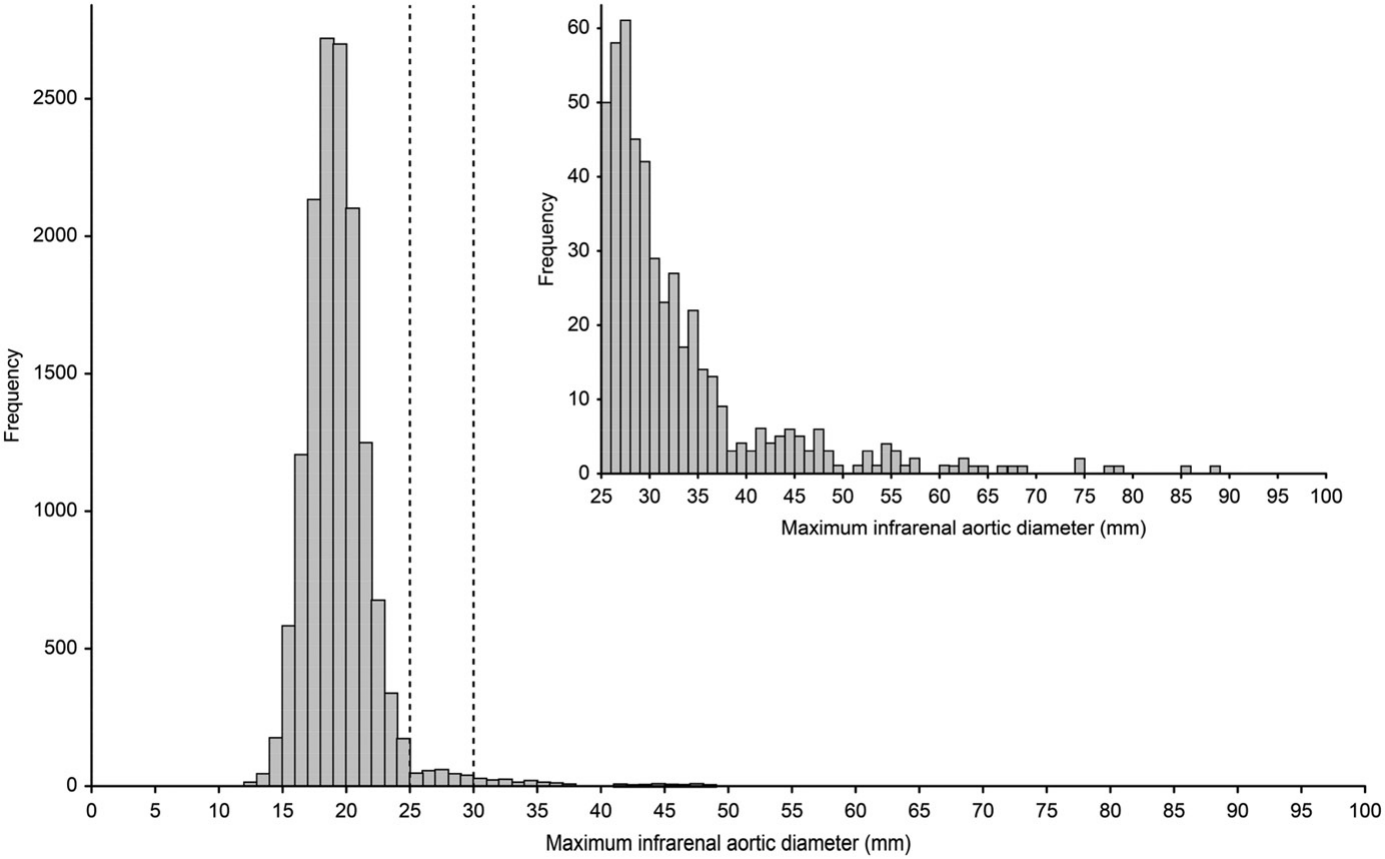
Infrarenal aortic diameters. Histogram presenting the distribution of the maximum infrarenal aortic diameter for the screened cohort of 65-year-old men. Embedded is a selective histogram of the size distribution of infrarenal aortic diameters ≥25 mm.

Of 14,678 distributed health questionnaires, 14,620 were completed (99.6%), which formed the cohort for further analyses. The following groups were based on the maximum anteroposterior diameter: normal aorta (<2.5 cm; *n* = 14,129), SAA (2.5–2.9 cm; *n* = 258), and AAA (≥3.0 cm; *n* = 233) (Figure 2). Current or former smoking status with a longer smoking duration, coronary artery disease, hypertension, hyperlipidemia, and claudication were substantially more frequently identified among those with SAA than among those with a normal aortic diameter. Notably, smoking duration was considerably longer in the AAA group than in the SAA group (*P* < 0.001) (Table 1); otherwise, the risk factor profile was very similar between the two groups. The distribution of risk factors associated with SAA and AAA compared with those associated with a normal aorta is shown in Table 1.

**Table 1.** Risk factors associated with subaneurysmal aorta (SAA), abdominal aortic aneurysm, and normal aorta in 65-year-old men.

Risk factor	Normal aorta (*n* = 14.129)	*P* value	Subaneurysmal aorta (*n* = 258)	*P* value	AAA (*n* = 233)
Ever smoked	63.0% (62.2–63.8)	<0.001	81.0% (76.2–85.8)	0.065	87.1% (82.8–91.5)
Current smoker	12.7% (12.2–13.3)	<0.001	28.7% (23.1–34.2)	0.30	33.0% (27.0–39.1)
Smoke-years	15.7 (15.4–16.0)	<0.001	24.8 (22.5–27.0)	<0.001	30.6 (28.3–32.8)
Pack-years	10.7 (10.4–10.9)	<0.001	17.2 (15.2–19.2)	<0.001	23.7 (20.9–26.6)
First-degree relative with AAA	1.4% (1.2–1.6)	0.22	2.3% (0.5–4.2)	0.39	1.3% (0.2–2.8)
Coronary artery disease	10.9% (10.4–11.4)	<0.001	19.8% (14.9–24.7)	0.11	25.8% (20.1–31.4)
Hypertension	36.8% (36.0–37.6)	<0.001	49.6% (43.5–55.8)	0.20	55.4% (48.9–61.8)
Hyperlipidemia	23.2% (22.6–23.9)	<0.001	36.0% (30.2–41.9)	0.38	39.9% (33.6–46.3)
Cerebrovascular disease	4.5% (4.2–4.8)	0.002	8.5% (5.1–12.0)	0.72	9.4% (5.7–13.2)
Claudication	1.2% (1.1–1.4)	<0.001	5.0% (2.4–7.7)	0.25	3.0% (0.8–5.2)
COPD	6.4% (6.0–6.8)	0.52	5.4% (2.6–8.2)	0.12	9.0% (5.3–12.7)
Diabetes mellitus	12.2% (11.6–12.7)	0.90	12.4% (8.4–16.5)	0.56	10.7% (6.7–14.7)
Renal insufficiency	0.9% (0.8–1.1)	0.82	0.8% (0.3–1.9)	0.18	0

AAA = abdominal aortic aneurysm; COPD = chronic obstructive pulmonary disease.

A multivariable logistic regression analysis (Table 2) identified current and ever smoking status, coronary artery disease, hypertension, hyperlipidemia, and claudication as independent risk factors for SAA, of which current smoking status yielded the highest OR (OR 2.8; 95% CI, 2.1–3.7; *P* < 0.001). In the AAA group, current smoking had a higher OR than in the SAA group (OR 3.5; 95% CI, 2.7–4.7; *P* < 0.001), and coronary artery disease, hypertension, and hyperlipidemia were also independently associated with AAA.

**Table 2.** Multivariable logistic regression analysis of covariables associated with subaneurysmal aorta (SAA) and abdominal aortic aneurysm (AAA), with the normal aorta group as the reference category.

Risk factor	SAA			AAA		
	Odds ratio	95% CI	*P* value	Odds ratio	95% CI	*P* value
Current smoker^a^	2.8	2.1–3.7	<0.001	3.5	2.7–4.7	<0.001
Ever smoked^a^	2.3	1.7–3.1	<0.001	3.6	2.4–5.3	<0.001
10 smoke-years^a^	1.3	1.2–1.4	<0.001	1.6	1.5–1.7	<0.001
10 pack-years^a^	1.2	1.1–1.3	<0.001	1.3	1.3–1.4	<0.001
Coronary artery disease	1.4	1.0–2.0	0.04	2.0	1.4–2.7	<0.001
Hypertension	1.3	1.0–1.7	0.03	1.6	1.2–2.1	0.001
Hyperlipidemia	1.4	1.0–1.8	0.03	1.4	1.0–1.9	0.03
Cerebrovascular disease	1.4	1.0–2.3	0.11	1.5	1.0–2.4	0.07
Claudication	2.5	1.4–4.6	0.003	1.1	0.5–2.5	0.7

The covariables with *P* < 0.1 in the univariate analysis were included in the multivariable regression analysis.

^a^Smoking covariables were entered separately into the analysis.

**Figure 3.**
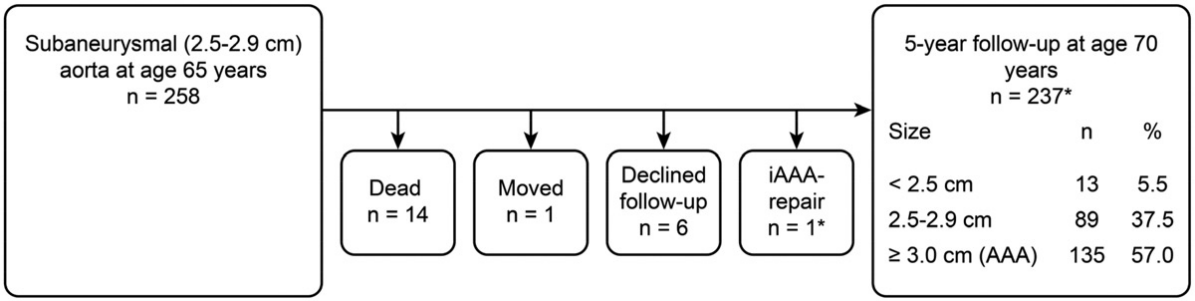
Flow chart of the SAA cohort. *One man underwent elective AAA repair after 4.5 years of follow-up for a large iliac aneurysm and a 4.5-cm iAAA and was included among the attenders. AAA = abdominal aortic aneurysm; iAAA = intact abdominal aortic aneurysm.

Of the 258 individuals with an SAA at baseline screening (Figure 3), a total of 14 (5.4%; 95% CI, 2.6–8.2) died of non-AAA-related causes within 5 years. A total of six men declined follow-up, and one man moved abroad. One man had undergone elective AAA repair for a large iliac aneurysm and a 4.5-cm AAA after 4.5 years of follow-up and was included in the attenders. A total of 237 (91.9%; 95% CI, 88.6%–95.2%) men were rescanned at 70 years of age. Among those re-examined at age 70, 13 (5.5%; 95% CI, 2.6%–8.4%) men had aortic diameters less than 2.5 cm. Eighty-nine (37.5%; 95% CI, 31.3%–43.75%) men were classified as still having an SAA (2.5–2.9 cm). A total of 135 (57.0%; 95% CI, 50.7%–63.3%) men had reached an aortic diameter of 3.0 cm or greater within 5 years after the initial scan. The annual mean expansion rate was 0.72 mm (95% CI, 0.60–0.84 mm). The frequency of smoking was consistently higher, the smoking duration was consistently longer (*P* < 0.001), and hyperlipidemia was more frequent (*P* = 0.031) among the subgroup of men with SAA expanding to AAA within 5 years than among those with SAA that did not expand to AAA. Regarding the risk factor profile, this subgroup displayed marked similarities with the AAA subgroup at baseline screening (Table 3).

**Table 3.** Risk factors for stable SAA versus expanding SAA during the 5-year follow-up.

Risk factor	SAA → aorta <3.0 cm (95% CI) (*n* =** **102)	SAA → aorta ≥3.0 cm (95% CI) (*n* = 135)	Odds ratio (95% CI)	*P* value
Ever smoked	74.5% (65.9–83.1)	85.2% (79.1–91.1)	1.97 (1.03–3.77)	0.040
Current smoker	22.0% (13.0–30.0)	36.0% (27.0–44.0)	2.01 (1.11–3.62)	0.019
Smoke-years	20.7 (17.5–23.9)	29.6 (26.6–32.6)	1.03 (1.02–1.05)	<0.001
Pack-years	15.1 (12.3–17.9)	22.1 (19.1–25.1)	1.03 (1.01–1.05)	0.002
First-degree relative with AAA	1.0% (0.0–2.9)	3.7% (0.5–6.9)	3.89 (0.45–33.78)	0.186
Coronary artery disease	18.0% (10.0–25.0)	24.0% (16.0–31.0)	1.45 (0.76–2.76)	0.258
Hypertension	46.0% (36.0–56.0)	53.0% (45.0–62.0)	1.34 (0.78–2.24)	0.269
Hyperlipidemia	31.0% (22.0–41.0)	45.0% (37.0–50.0)	1.80 (1.05–3.09)	0.031
Cerebrovascular disease	8.0% (3.0–13.0)	10.0% (5.0–16.0)	1.36 (0.55–3.38)	0.507
Claudication	2.0% (0.0–5.0)	5.0% (1.0–9.0)	2.73 (0.56–13.55)	0.198
COPD	5.0% (1.0–9.0)	4.0% (1.0–8.0)	0.90 (0.27–3.04)	0.868
Diabetes mellitus	17.0% (9.0–24.0)	10.0% (5.0–15.0)	0.53 (0.25–1.16)	0.107
Renal insufficiency	2.0% (0.0–5.0)	0	0.43 (0.37–0.49)	0.102
Antiplatelet use	26.0% (1.8–35.0)	33.0% (25.0–41.0)	1.39 (0.79–2.45)	0.255
Statin use	31.0% (22.0–41.0)	41.0% (33.0–50.0)	1.55 (0.90–2.66)	0.111

AAA = abdominal aortic aneurysm; SAA = subaneurysmal aorta.

## Discussion

In this cross-sectional population-based study, the prevalence of SAA among 65-year-old men in middle Sweden was 1.9%, which was similar to the proportion of men with AAA (1.7%). The prevalence of SAA was slightly lower than that observed in other populations (11,13,15). It should be noted that 2.6 cm was used as the lower threshold for this particular subgroup in the Gloucester study, with a prevalence of 2.0% compared to 2.5 cm in the present study (13). A higher prevalence was reported by Duncan et al. (23), (8.2%), but that study included a wider age range of men (65–74 years).

The main finding in the present study is the marked similarity in the risk factor profile of men with SAA and men with AAA. The most important risk factor for both SAA and AAA is smoking, with the highest OR for current smoking, which was also associated with more extensive aortic pathology, especially among men with SAA progressing to AAA within 5 years. The observed association between smoking and SAA as well as between smoking and AAA is consistent with the findings of previous studies (14,17–19,22). The observed incremental association between smoking duration and disease severity in the present study strongly suggests a dose-response relationship.

This indicates that SAA and AAA may have the same pathophysiological origin and that SAA should be considered as an early stage of aneurysm formation, as suggested by several authors (11–14,22,27). Cohort and observational studies have shown that SAA expands to a large extent to true AAA and may rupture over time (5,15,16). Svensjö et al. (14) showed in the Uppsala cohort that an infrarenal aortic diameter of 2.5–2.9 cm and smoking were the most important risk factors for the development of AAA within 5 years. The presence of an SAA at 65 years of age resulted in a 60-fold increased risk of AAA formation.

A meta-analysis from the RESCAN collaborators concluded that smoking increased the yearly expansion rate of AAAs by 35% and doubled the rupture risk. One limitation was that the data were strictly limited to aortic diameters of 3.0–5.4 cm (28). A negative correlation between smoking cessation and the progression of AAAs has also been observed (29). Smoking cessation is to date the only known effective interventions to prevent small AAAs from further expansion and rupture and has been shown to be highly cost-effective (30,31). It is reasonable to assume that subjects with SAA could also benefit from targeted smoking cessation programs, and smoking cessation strategies targeting this subgroup should be considered (32). This requires, however, that the SAAs are detected, which to a great extent is only possible through population screening.

One of the consequences of implementing screening programs is the detection of a considerable number of small AAAs and SAAs. There are still uncertainties and substantial variations in the surveillance recommendations for aortas with diameters of 2.5–2.9 cm (SAA) (9). Based on the design of the RCTs, many screening programs consider this subgroup not to be significant and conclude that further surveillance or interventions are unnecessary (33,34), whereas others consider SAAs to be abnormal and recommend surveillance (11–14,22,27). The 2019 European Society for Vascular Surgery (ESVS) Clinical Practice Guidelines on the Management of Abdominal Aorto-iliac Artery Aneurysms issued a weak recommendation to re-screen men with a SAA after 5–10 years (35).

In this observational population-based study with a high attendance rate, we report on a cohort with screening-detected SAAs, which presented a high rate of progression to true AAAs after 5 years of follow-up. Thirteen (5.5%) men with a previous SAA were classified as having a normal aorta (<2.5 cm) at rescanning. Six patients had an aortic diameter of 2.5 and 2.6 cm at baseline screening. The most likely explanation was that the aortic diameter was over- or underestimated due to variability in the US measurement technique within the standard deviation (SD), i.e. limits of agreement. A recent study demonstrated that the LELE method used in the present study has a variability of 2 mm (25). The remaining seven men were considered to have been misclassified due to measurement error at the baseline screening.

In a multicenter observational study by Wild et al. (15) that included 1696 individuals with SAA from eight European screening programs, 67.7% of the subjects progressed to an AAA after 5 years of surveillance, and a total of 26.2% of this subgroup had reached the threshold for repair of ≥5.5 cm after 10 years of follow-up. Similar findings were also evident in a Swedish longitudinal cohort study in which 3268 men were rescanned 5 years after baseline screening at 65 years of age. In total, 52.2% of subjects with SAA had progressed to AAA within 5 years (14). A recent publication from the Gloucestershire Aneurysm Screening Programme that included 1233 men with SAA estimated that 57.6% would progress to AAA 5 years after the initial scan and that 28% would develop a large AAA by 80 years of age (16). Long-term follow-up data from the MASS randomized trial showed the occurrence of ruptures after 8 years among men initially screened as normal (<3 cm). More than 50% of these ruptures occurred among men with an aortic diameter of 2.5–2.9 cm at the baseline screening (5). Ruptured AAA is, to date, more common in subjects older than 75 years of age, and the expectation for intervention in older patients has increased (7,36,37). Rapid acquisition of minimally invasive technologies, such as endovascular aneurysm repair (EVAR), and the ability to intervene have also increased (38). Thus, there is an urgent need for the development of evidence-based strategies as to whether the subgroup of individuals with SAA should be monitored or not (39). This is even more relevant now that we see an ever-increasing longevity in the population (12,14,20). There is, however, only limited evidence regarding the clinical relevance and cost-effectiveness of surveillance of persons with SAA (40). There are also psychological aspects that need to be evaluated, to ensure that monitoring of the SAA does not do more harm than good.

The implementation of population-based AAA screening programs in Europe and the USA has coincided with a significant change in the epidemiology of the disease: 1) decreased incidence, mainly due to reduced smoking rates; 2) altered management, most importantly the introduction of EVAR with improved outcomes and more patients being offered treatment; and 3) increased longevity in the general population (12,14,20,39). Model studies have demonstrated that the observed decrease in prevalence is counterbalanced by decreased perioperative mortality and increased longevity, resulting in an unchanged low cost per quality-adjusted life-year (QALY) gained (41). This conclusion was confirmed in a recent report from the Swedish nationwide AAA screening program, showing a 27% reduction in AAA mortality after 10 years of screening, with an incremental cost-efficiency ratio of €7,770 per QALY gained, corresponding to €49,800 per life saved from rupture. Although screening for AAA remains highly cost-effective in a contemporary setting, the effectiveness is less than previous calculations based on older RCTs with a higher prevalence of the disease (12). An expanded surveillance program that includes follow-up of SAA with smoking cessation and secondary cardiovascular prevention programs in this subgroup might have the potential to further improve the effectiveness of AAA screening programs but needs further evaluation regarding the long-term effects, including health-economy and aspects on quality of life.

In conclusion, our findings demonstrated a marked similarity in the risk factor profile between men with SAA and men with AAA. Smoking was the most important risk factor, and there was an incremental association between smoking duration and disease severity, especially among men with SAA progressing to AAA within 5 years. This finding indicates that SAA and AAA may have the same pathophysiological origin and supports that SAA should be considered an early stage of aneurysm formation. Further research on the cost-effectiveness and potential benefits of surveillance as well as smoking cessation and secondary cardiovascular prevention programs in this subgroup is warranted.
